# Directional biases in whole hand motion perception revealed by mid-air tactile stimulation

**DOI:** 10.1016/j.cortex.2021.03.033

**Published:** 2021-09

**Authors:** Marlou N. Perquin, Mason Taylor, Jarred Lorusso, James Kolasinski

**Affiliations:** a Cardiff University Brain Research Imaging Centre, School of Psychology, Cardiff University, UK; b Biopsychology & Cognitive Neuroscience, Faculty of Psychology and Sports Science, Bielefeld University, Germany; c Cognitive Neuroscience, Faculty of Biology, Bielefeld University, Germany; d School of Biological Sciences, University of Manchester, Manchester, UK

**Keywords:** Haptics, Somatosensory, Touch, Human–computer interaction, Confidence

## Abstract

Many emerging technologies are attempting to leverage the tactile domain to convey complex spatiotemporal information translated directly from the visual domain, such as shape and motion. Despite the intuitive appeal of touch for communication, we do not know to what extent the hand can substitute for the retina in this way. Here we ask whether the tactile system can be used to perceive complex whole hand motion stimuli, and whether it exhibits the same kind of established perceptual biases as reported in the visual domain. Using ultrasound stimulation, we were able to project complex moving dot percepts onto the palm in mid-air, over 30 cm above an emitter device. We generated dot kinetogram stimuli involving motion in three different directional axes (‘Horizontal’, ‘Vertical’, and ‘Oblique’) on the ventral surface of the hand. Using Bayesian statistics, we found clear evidence that participants were able to discriminate tactile motion direction. Furthermore, there was a marked directional bias in motion perception: participants were both better and more confident at discriminating motion in the vertical and horizontal axes of the hand, compared to those stimuli moving obliquely. This pattern directly mirrors the perceptional biases that have been robustly reported in the visual field, termed the ‘Oblique Effect’. These data demonstrate the existence of biases in motion perception that transcend sensory modality. Furthermore, we extend the Oblique Effect to a whole hand scale, using motion stimuli presented on the broad and relatively low acuity surface of the palm, away from the densely innervated and much studied fingertips. These findings highlight targeted ultrasound stimulation as a versatile method to convey potentially complex spatial and temporal information without the need for a user to wear or touch a device.

## 1 Introduction

The human tactile system is increasingly being targeted as a means to communicate explicit information via spatial and temporal stimuli from electronic devices. This trend has developed from a desire to avoid saturating the auditory and visual systems. Although such tactile-based technologies have made rapid advancements, the theoretical understanding of how we process and perceive these tactile stimuli remains largely behind.

**Fig. 1:**
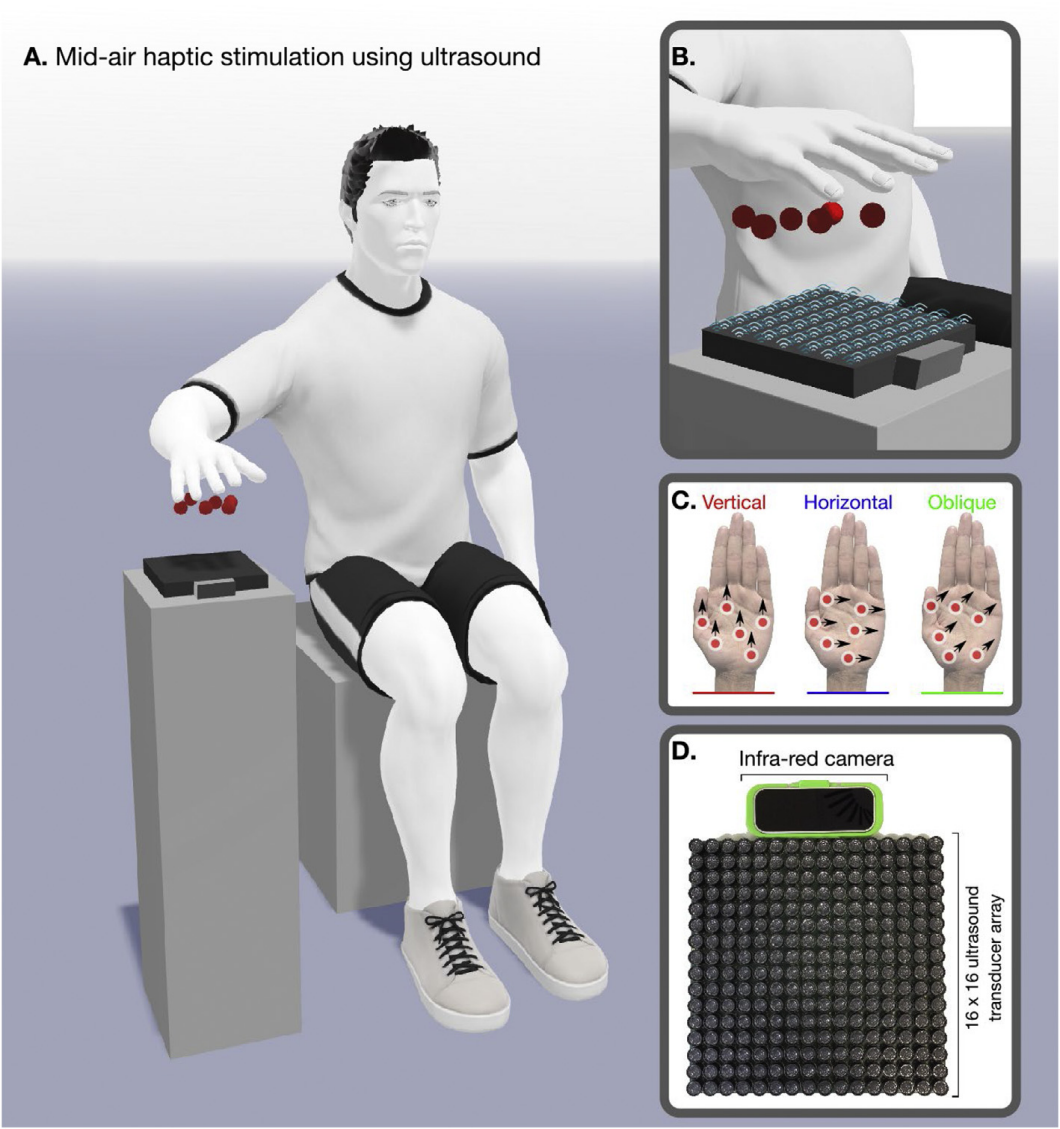
**Overview of mid-air tactile experimental setup**. A. Participants were seated with their hands above an array of ultrasound actuators and a infra-red camera. B. The combination of real time hand tracking and focused ultrasound can project discrete points onto the user's unadorned hand (Carter et al., 2013). C. Users experienced a series of moving dot stimuli (Fig. 2) in differing directions. D. Stimuli were delivered using an Ultrahaptics device (UltraLeap, Bristol).

In particular, the recent development of targeted ultrasound technology has made it possible to project complex tactile percepts or ‘scenes' onto the surface of the hand without any physical contact (Carter et al., 2013) (see Fig. 1). This technology can produce stimuli translated almost directly from the visual domain, including defined points, lines, and shapes; both static and moving. Mid-air touch stimulation uses an array of ultrasound emitters and infra-red hand tracking to deliver stimuli with a high spatial and temporal frequency, targeted to specific regions of the palmar surface of the hand, up to 80 cm above an emitter device. Just as light is projected onto the retina for vision, ultrasound technology can project tactile scenes directly onto the hand.

The development of such advanced stimulation technology has arguably outstripped our understanding of human tactile perception. Of particular importance is the question of whether the hand can be used to perceive the relatively complex stimuli that is it now feasible to produce. The motivation for developing such technologies is predicated on the assumption that humans can perceive the spatial and temporal features of whole hand tactile stimuli in the same way we would perceive equivalent visual stimuli with our eyes. Although the spatial and temporal features of such whole hand tactile stimuli prompt obvious parallels with visual stimuli (shapes, lines, motion), touch is a distinct sensory domain, processed in distinct cortical regions (Gardner, 1988; Pei et al., 2010, 2011; Tamè et al., 2019).

Studies of human touch sensation on the hand have been dominated by investigation of the fine-grain perceptual abilities of small regions of high tactile acuity at the fingertips (Mountcastle, 2005). However, studies building on Weber's illusion have demonstrated spatial distortions in the perception of distance on the hairy skin of the dorsal hand, but not on the palmar glabrous skin (Cody et al., 2008; Knight et al., 2014; Longo & Haggard, 2011). Recent studies have reported the existence of spatial biases on the glabrous skin of the palm, with the orientation of tactile stimuli impacting the perception of time (Hidaka et al., 2020) and distance (Fiori & Longo, 2018) - though to a smaller magnitude compared with the hairy skin. Still, this reduced anisotropy might be good news for tactile communication technologies targeting the palmar area - although this of course remains highly speculative for now.

If the palm of the hand can indeed be used to perceive complex stimuli similar to vision, one may also ask if it exhibits the same kinds of well-documented perceptual biases reported extensively in the visual domain. The presence of such common biases across the visual and tactile modalities would further support the notion of common mechanisms of multi-sensory processing in the human brain (Murray et al., 2016). An understanding of the biases in whole hand tactile function is also essential to the development of tactile technologies that work in synergy with human perceptual abilities. A much-studied example of a visual perceptual bias is the oblique effect (Appelle, 1972): the well-established phenomenon that perception of motion or orientation in horizontal and vertical axes is superior to that in intermediate oblique axes (e.g., Ball & Sekuler (1980); Ball & Sekuler, (1987)).

Here we translate a classic visual dot kinetogram stimulus into the tactile domain using focused ultrasound stimulation. We investigate the existence of the oblique effect in the tactile domain on the palm in stimuli presented vertically (proximal-distal axis), horizontally (medial-lateral axis), and obliquely between these two cardinal planes. In applying highly novel ultrasound technology to tactile psychophysics, we address two specific questions: (1) can the human hand can be used to accurately and confidently perceive the complex dot motion stimuli in the tactile domain, and (2), does the oblique effect manifest in the perception of tactile motion stimuli presented in different orientations on the palm?

## 2 Materials and methods

We report how we determined our sample size, all data exclusions (if any), all inclusion/exclusion criteria, whether inclusion/exclusion criteria were established prior to data analysis, all manipulations, and all measures in the study.

### 2.1 Participants

Fourty-five participants (29 female, age range: 19–40, Mean_age_ = 24.2) were tested in a two-day experiment. Participants were right-handed, with no self-reported touch deficits in their hands or upper limbs, and were paid 30 pounds in total for participation. The study was approved by the local ethics committee (Cardiff School of Psychology Research Ethics Committee: EC.18.06.12.5311R). Three participants were excluded from analysis: the first responded almost exclusively with one response key, and the other two were excluded because of repeated non-compliance to instructions to keep their hands above the tactile device and to keep their heads in the chin rest - which led to a high number of experiment crashes and very long pauses between trials (as observed by the experimenter during testing).

We aimed to collect minimally twelve participants and then further collect until a Bayes Factor of 6 (either for or against an effect of condition) was reached (Rouder, 2014; Schönbrodt et al., 2017). With twelve participants, we had already reached this threshold (Fig. 3B). However, although the overall patterns in the data were clear, differences between individuals were large. We therefore opted to test more participants, to be able to examine these differences systematically. Because we had no prior expectations, no new threshold was set for the second batch of collection, but instead was tied to an undergraduate project, and was stopped once the undergraduate practical data collection requirements were met.

### 2.2 Materials

**Fig. 2:**
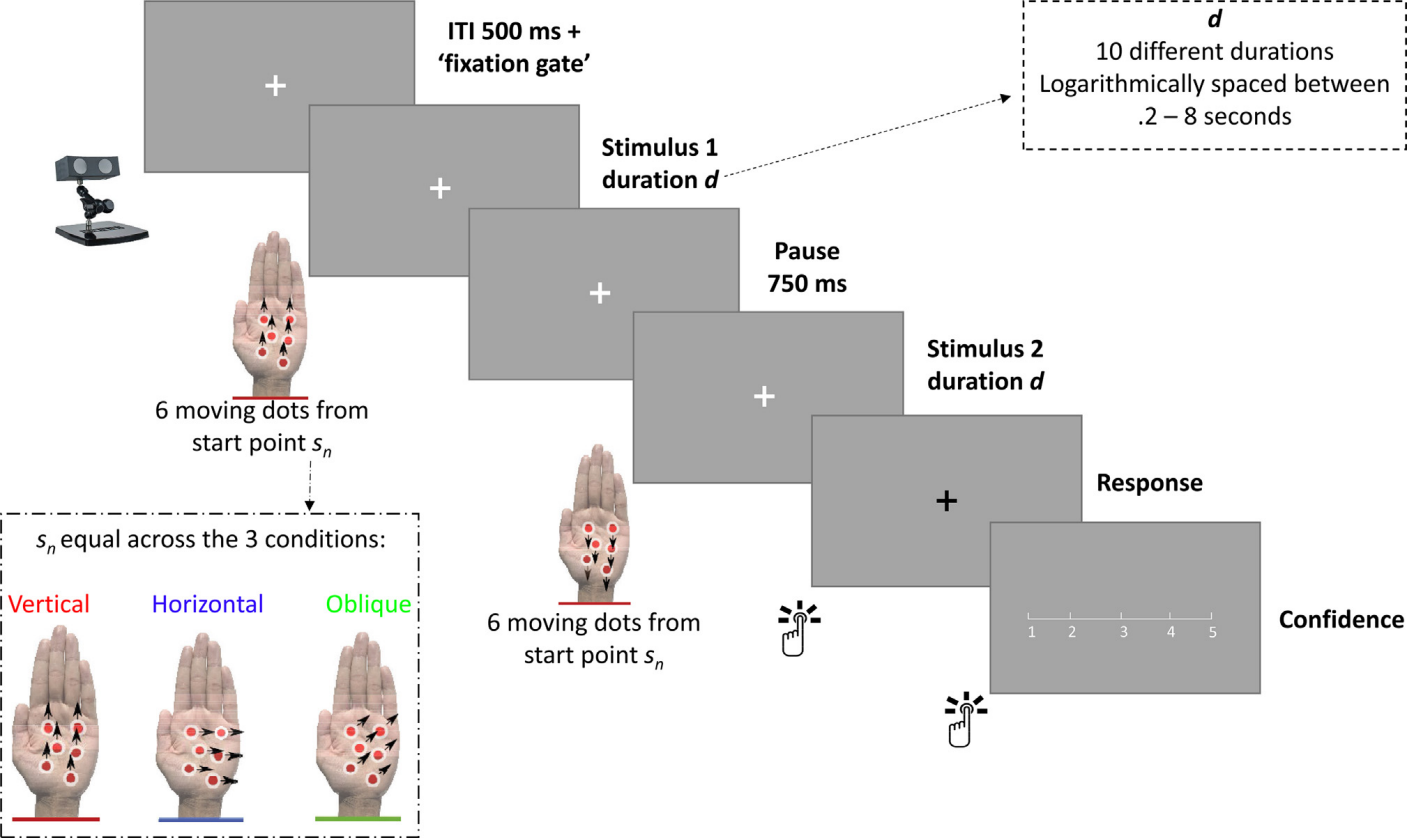
Overview of a single experimental trial presenting mid-air tactile stimuli in a two-interval-forced-choice task.

The experiment was generated using PsychoPy 3.2 (Peirce, 2007, 2009; Peirce et al., 2019) and Visual Studio 16 (Microsoft, Redmond, US), run on a Viglen Vig800S computer (Viglen Ltd, Hertfordshire, UK). The tactile stimuli were generated from an UltraHaptics UHEV1 Array (UltraLeap, Bristol, UK), which was attached with a Leap Motion camera for continuous hand-tracking. The visual instructions and elements of the experiment (Fig. 2) were displayed on an ASUS VG248QE monitor (resolution: 1920 × 1080; refresh rate: 144 Hz; AsusTek Computers Inc, Taipei, Taiwan). Participants’ eyes were tracked with a LiveTrack Lightning Eye Tracker (Cambridge Research Systems Ltd, Kent, UK). Responses were recorded with a 5-button NAtA Technologies Response Box (NAtA Technologies, Coquitlam, Canada). Hand and body temperature were recorded with an NC200 Non Contact Forehead Thermometer (Medisave UK Ltd, Weymouth, UK). 3D visualisations of the experimental paradigm were generated using MagicPoser (Wombat Studio, Inc., Santa Clara, California, US).

**Fig. 3:**
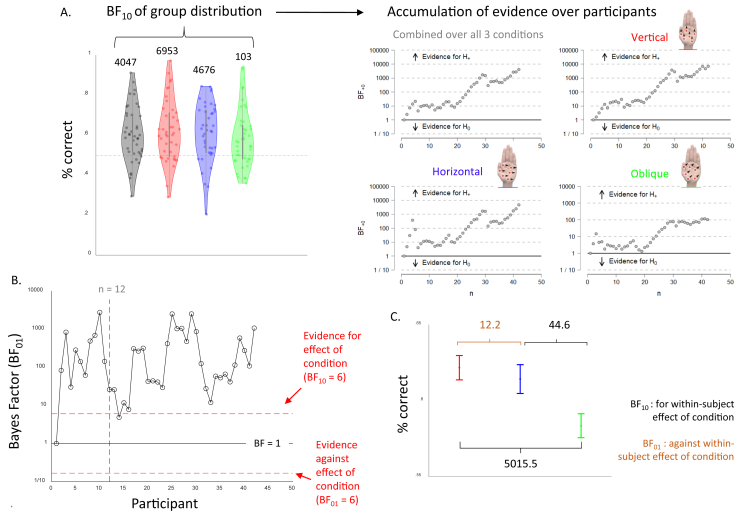
**Evidence of an oblique effect in whole hand tactile motion perception.** Performance on the experimental two-interval-forced-choice task for the three conditions combined (grey) as well as separate for vertical (red), horizontal (blue), and oblique (green) tactile motion stimuli presented on the palm. A. Group distributions of % correct (left), with each dot in a distribution showing the performance of one participant, with the accompanying BF_10_ from the one-sample *t*-tests presented above. The BF_10_ show extreme evidence that % correct exceeds chance level. The right panel shows the change in BF_10_ as a function of participant recruitment, reflecting the accumulation of evidence as the sample size increased. B. Bayes Factor (BF_inclusion_) of effect of condition a function of participant recruitment, showing the cut-offs for the minimally to be collected sample size (n = 12 or BF_inclusion_ for condition > 6) C. Plot of the group mean of each condition with the within-subject error bars - reflecting the within-subject differences across the three conditions - with the BF_10_ of the post-hoc tests above. Post-hoc tests indicate a clear existence of an “Oblique” effect in the data, such that participants performed statistically better in perceptual discrimination in the horizontal and vertical axes compared with the oblique axis.

### 2.3 Design

Each participant took part in a two-interval-forced-choice task, in which they were instructed to discriminate tactile motion direction. This was undertaken in three different conditions (Fig. 2): 1) horizontal (with stimuli moving along the 90–270°axis, along the medial-lateral axis of the palm; one stimulus would move to the left, and the other stimulus would move to the right), 2) vertical (0–180°axis, along the proximal-distal axis of the palm), and 3) oblique (45–225°axis). After the presentation of the two stimuli in a given axis, participants judged which of the two stimuli respectively moved: 1) rightwards, 2) downwards, and 3) oblique downwards.

All stimuli consisted of 6 tactile dots (8.5 mm diameter), moving coherently at a speed of 4 cm/s. Dots were selected as they do not provide any other potential motion cues, such as shape and orientation. The area on the palm of the hand in which the motion occurred extended across the full medial to lateral extent of the palmar surface. The proximal-distal extent of the palmar motion area was equal to the medial to lateral width of the palmar surface extending from the heel of the hand to the proximal aspect of the fingers.

The six tactile dots were generated by the Ultrahaptics array starting from *s*_n_, with *s* being a randomly-assigned start point at trial *n*. Note that *s*_n_ was kept equal over the three direction conditions (i.e., for one participant, the start position of the six random dots on trial *n* were the same in each condition). In addition, motion was limited such that the moving dots would not extend beyond the palmar area (meaning participants would always be able to feel motion for the full stimulus duration). The Leap camera combines a stereo infra-red camera with tracking algorithms to reconstruct a 3D model of the hand from the raw sensor data, with a stated accuracy from the manufacturer of .01 mm (Weichert et al., 2013). Any dots extending beyond the motion area on the palm were re-generated at a pseudorandom position in the motion area, but were prevented from overlapping with the position of any existing dot. So for example, during a trial with an upwards motion, if a dot reached the end of the hand, it was relocated to a new position on the hand on which it would not overlap with any of the other five dots. In trials with longer durations, the stimuli hence felt as a kind of continuous flow. The displacement induced by ultrasound has been visualised using oil bath preparations (Abdouni et al., 2019), however, no such estimates regarding the magnitude of displacement have been made on human skin; based on microneurography studies of brief air puff stimuli, it is conceivable that ultrasound stimuli may excite both rapidly and slowly adapting receptor subtypes (Mizobuchi et al., 2000).

Previous pilot studies using Ultrahaptics (Korres and Eid, 2016; Rutten et al., 2019) have selected only very long stimulus durations, respectively lasting 9 and 30 sec. As the consequences of such long durations are still unknown, we presented stimuli across a broad range of 10 different durations *d*, which were logarithmically spaced between 200 msec and 8 sec, allowing us to investigate the potential for accurate perception across a wide variety of exposures. These different durations were manipulated across trials (i.e., within each trial both stimuli always had the same duration).

### 2.4 Procedure

Participants visited the lab for two sessions, each 1–1.5 h in length. In the first session, they completed the Edinburgh Handedness Inventory (Oldfield, 1971) to verify their right-handedness. Next, they were seated in a chin-rest, 55 cm from the screen. Their right arm was immobilised up until their wrist on an arm-rest with velcro straps to eliminate motion, with their hand 38 cm above the Ultrahaptics device. In this set-up, participants were only able to partially move their hand ‘down’ (at an ∼45° angle from the arm) and up. Within blocks, they were instructed to keep their hand as still and straight as possible. To block any auditory cues from the presented stimuli, participants were given 35 dB ear plugs as well as a pair of 27.6 dB ear defenders. During the training period participants wore the ear defenders without ear plugs to allow for verbal discussion and questions.

Participants first performed a training block. Next, they were taken out of the arm-rest, to ask any questions and to fit ear plugs. After the training, the eye tracker was calibrated using a 9-point paradigm. Participants subsequently performed two experimental blocks of each condition (horizontal, vertical, and oblique). In the second session, participants performed three more experimental blocks per condition - resulting in five experimental blocks in total.

In the training block, each trial started with a white fixation cross, presented for 500 msec. The first stimulus was then delivered for 1 sec, followed by a 750 msec pause, and then the second stimulus was delivered for 1 sec. After stimulus presentation, the fixation cross turned black, prompting a response from the participant. After a correct response, the cross turned green, and after an incorrect response, the cross turned red. Participants completed six trials for each condition, with a self-passed break in between each condition.

The experimental blocks, which comprised the majority of the testing, mirrored the training blocks aside from three key differences. Firstly, participants were not given feedback after their responses, but instead were asked to rate the confidence in their answer on a scale from 1: “not at all confident”) to 5: “completely confident” (Fig. 2) with a button press using the response box. Secondly, the stimulus duration was varied across trials at intervals logarithmically spaced between 200 msec and 8 sec. Finally, each trial included a ‘fixation gate’ - a gate for which participants had to continuously fixate for ∼175 msec (25 frames) before the trial would begin - presented after the initial fixation dot of 500 msec. Note that participants did not receive explicit instructions on this; they were instructed to keep fixation throughout the trial. If they did not fixate, the trial would not yet start. This fixation gate was not added to control for potential effects of eye gaze, as previous literature has suggested that tactile perception improves when looking at the respective body part (Kennet et al., 2001; Taylor-Clarke et al., 2002). Though it remains speculative if looking at the hand would improve whole-hand perception with ultrahaptic stimuli, our current fixation gate would keep any potential effects consistent over trials. Furthermore, the fixation gate ensured that participants were attentive prior to the start of the trial, which was important considering the length of the experiment and the requirement to sit very still throughout blocks. Exploratory correlational analyses after data collection showed that eye gaze was not correlated with discrimination accuracy, neither on the within-subject level (participants’ accuracy on a trial did not depend on how well they were fixating during that trial) nor on the between-subject level (participants who fixated more did not have different accuracy levels on average) - analyses and data available on OSF.

Each experimental block consisted of 40 trials, with 4 times each of the 10 durations, randomly presented throughout block (but see section: Technical issues); each block comprised only one condition. There was a self-paced break after every 10 trials, and a longer break between each condition - this large number of breaks was included to minimise discomfort and to reduce participant motion during the trials. We were unsure if any learning effects in discrimination performance would occur over time or across conditions. Therefore, conditions were presented in a blocked order (keeping any potential learning effects steady across conditions within participants), and orders were counterbalanced across participants both in training and experimental phase (to prevent systematic differences in learning effects between conditions on the group level). We discuss potential training effects over experimental blocks further in the Results section.

During pilot studies preceding the current experiment, large differences in performance and confidence were already apparent between participants. Aiming to explain these differences, we measured a number of candidate variables that may affect tactile performance. At the beginning of both sessions, body temperature (measured on the forehead and the right hand), hand size (measuring from the wrist to the tip of the middle finger), and (middle) finger length were also measured. We also considered task effects on performance, namely (between-subject) condition order and (within-subject) time on task (i.e., block number).

#### 2.4.1 Technical issues

A common technical issue we experienced was the Ultrahaptics emitter crashing within a trial - possibly because the device is not specifically made for experimental purposes, in which it is necessary to run a large number of trials in quick succession. To limit the amount of these crashes, we reconnected the device after every 40-trial block. However, we were not able to prevent the crashes altogether, and still had 157 crashes over all sessions combined (3.7 crashes per participant on average, SD = 2.8, range 0–12). Wherever possible, participants performed extra trials, aiming to achieve a *minimum* of 200 trials in each condition (total trial mean across 3 conditions = 614, SD = 25, range: 560 to 660). Number of trials did not vary systematically between condition, BF_01_ = 12.5.

### 2.5 Data preparation and analysis

Training trials were excluded from all analyses. Analyses were conducted in Matlab 2019a (MATLAB, 2019) and JASP (JASP Team, 2019). As this is the first study to systematically investigate the feasibility of tactile mid-air perception, we chose to use Bayesian statistics - allowing for assessment of both the alternative and the null hypothesis. Bayesian statistics were estimated using equal prior distributions and 10,000 iterations for Monte Carlo simulations. Note that no part of the study procedures and analyses have been preregistered prior to data collection.

#### 2.5.1 Performance

##### 2.5.1.1 Feasibility of tactile mid-air perception

Participant means for performance (% correct) were calculated for each of the three conditions separately, as well as combined over all conditions. Bayesian one-sample *t*-tests were conducted on the group distributions, to test if they were higher than chance level (50% correct). Because we had a clear directional expectation, these tests were conducted one-sided.

##### 2.5.1.2 Testing within-subject effects

Participant means were calculated separately for each condition and duration. A Bayesian 3 × 10 Repeated Measures (RM) ANOVA was conducted, with condition and duration as independent factors.

#### 2.5.2 Confidence

To assess participants' subjective experiences of the tactile stimuli, confidence ratings were measured after every trial. These ratings reflect how confident the participant actually felt in their ability to discriminate the stimuli. However, raw confidence ratings cannot quantify how *accurate* the participant is at judging their own performance (i.e., high ratings for correct responses, and low ratings for incorrect responses). Therefore, we also computed a measure of ‘meta-cognitive ability’, which reflects how much these subjective rating matches their objective performance. To assess such meta-cognitive ability, we estimated the type-II area under the receiver-operating curve (AROC) (Fleming et al., 2010; Fleming & Lau, 2014). This measure reflects how much the subjective ratings and performance ‘match’ given the number of possible values on the confidence scale. Together, the raw ratings and the AROC thus reflect two different pieces of the same puzzle.

##### 2.5.2.1 AROC: Quantifying meta-cognitive ability

Just as in behaviour, one could distinguish *hits* (in this case, a high confidence rating when the response is correct) from *misses* (a high confidence rating when the response is incorrect). To make such a distinction, one needs a criterion to determine if the rating is ‘high’ or ‘low’. To estimate the ROC, the proportion of hits can be plotted against the proportion of misses along all possible criteria (that is, the proportion calculated under low = 1 and high = 2–5, the proportion calculated under low = 1–2 and high = 3–5, and so on). Just as with typical ROCs, the area under the curve can then be quantified, giving the AROC measure. A key benefit of the type-II AROC is that it does not assume the confidence ratings follow a normal distribution - an assumption that is not met in the current data.

Chance level of the AROC measure is indicated by a value of .5. To assess whether the current values exceeded this level, Bayesian one-sided one-sample *t*-tests were conducted on the group distributions of AROC values for all three conditions plus combined.

##### 2.5.2.2 Testing within-subject effects

Mean confidence and AROC were calculated per participant separately for each condition and duration. Two Bayesian 3 × 10 Repeated Measures (RM) ANOVAs were conducted using condition and duration as independent factors.

#### 2.5.3 Examining inter-individual differences and task-effects

##### 2.5.3.1 Between-subject correlations

Participants' age and palm size (calculated as hand size - finger size) were linearly correlated between individuals to mean accuracy, confidence, and AROC (looking at combined, horizontal, vertical, and oblique trials over the entire session) - resulting in distributions of 2 between-subject variables (age and palm size) × 3 measures (accuracy, confidence, and AROC) × 4 ‘conditions’ (horizontal, vertical, oblique, and combined) = 24 correlation coefficients plus accompanying Bayes Factors. Bayesian ANOVAs with condition order as independent variable were calculated on the same outcome measures - giving 12 additional Bayes Factors.

##### 2.5.3.2 Within-subject correlations

To assess within-subject factors, accuracy, confidence, and AROC were calculated for each of the five experimental blocks, independently for combined, horizontal, vertical, and oblique trials. For each participant, these means were correlated to: 1) hand temperature prior to each block, 2) hand temperature before each block, corrected for body temperature, and 3) block number. This resulted in 3 within-subject variables (hand temperature, hand-body temperature, and block number) × 3 measures × 4 ‘conditions’ = 36 correlation coefficients per participant. We tested whether each of these coefficients were statistically different from zero on the group level, using Bayesian one sample *t*-tests.

#### 2.5.4 Interpretation Bayes Factors

BF_10_ represents the likelihood of the current data under the alternative (e.g., effect of condition) over the null hypothesis (e.g., no effect of condition). It is a continuous measure of evidence that can take any value between zero to infinity (Wagenmakers et al., 2018a). Note that the evidence for the null over the alternative hypothesis (BF_01_) is equal to the inverse of BF_10_. BF_10_ values above 1 indicate more evidence for the alternative hypothesis, while values under 1 indicate more evidence for the null-hypothesis - though as a rough rule of thumb, BF_10_ between $\frac{1}{3}$ and 3 are typically interpreted as ‘indeterminate evidence’ (Lee & Wagenmakers, 2013).

Bayesian RM ANOVA is a form of model comparison - assessing how much more likely the data is under the statistically-best model as compared to under each of the other models (Wagenmakers et al., 2018b). The output provides an ‘Analysis of Effects’, with BF_inclusion_ reflecting the average over all the models which include that factor; this is therefore the most comparable to ‘classic’ RM ANOVA within-subject effects. The Bayesian RM ANOVA also provides Bayes Factors to compare the models directly. In our current analyses, the model comparisons and the analyses of effects led to the exact same conclusions in each instance. Therefore, we chose to only report the BF_inclusion_. The Bayes Factors for model comparison, as well as all other analyses, can be found on the annotated.jasp files on OSF.

## 3 Results

### 3.1 Performance

#### 3.1.1 Discrimination of complex tactile percepts exceeds chance

On the group level, there was extreme evidence that performance was statistically above chance in all conditions (Fig. 3A), indicating that participants were able to distinguish the direction of complex tactile motion perceptions delivered across the palm. The highest evidence for performance above chance was observed in the vertical condition and the lowest was observed in the oblique condition. Fig. 3A shows the accuracy mean for each participant for both average performance and separately over the three conditions, with accompanying BF_10_ above. Sequential analyses reveal the trajectory of evidence accumulation across participant recruitment, reflecting the change in BF_10_ as the sample size increased. Average overall accuracy across all stimulus durations was not very high (overall group mean = 60.6%) and between-subject variance was high.

#### 3.1.2 Clear evidence of an oblique effect in tactile motion perception

The BF_inclusion_ indicate that there is extreme evidence for an effect of condition only. As shown in Fig. 3B, we reached our pre-specified threshold for the BF_inclusion_ after collecting the first batch of twelve participants. Fig. 3C shows the within-subject differences between the three conditions, with accompanying statistics in Table 1.

**Table 1:**
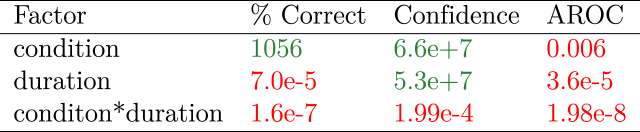
Overview of the BF_inclusion_ for the three independent factors - condition, duration, and their interaction - resulting from the three RM ANOVAs conducted on performance (% correct), confidence rating, and metacognitive ability (AROC). BF_inclusion_ that indicate evidence in favour of an effect (>3) are shown in green; BF_inclusion_ that indicate evidence against an effect (<$\frac{1}{3}$) are shown in red.

Post-hoc tests conducted on condition (Bayes Factors shown in Fig. 3B) showed that accuracy was lower in the oblique compared to the vertical and horizontal condition, with no difference between the horizontal and vertical conditions. The results indicate that participants performed significantly better in perceptual discrimination of tactile motion presented along the horizontal and vertical axes compared with the oblique axis, consistent with the notion of an oblique effect from the visual literature.

### 3.2 Confidence in tactile perception also shows oblique effect

**Fig. 4:**
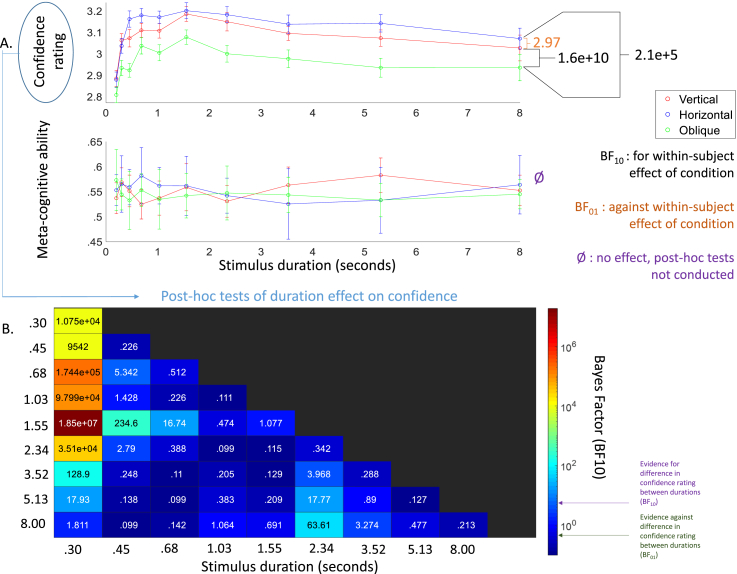
**Overview of results of the meta-cognitive measures**. A. Mean confidence rating (top panel) and mean meta-cognitive ability (AROC; bottom panel) for the vertical (red), horizontal (blue), and oblique (green) condition over the ten different stimuli durations (logarithmically spaced between 200 msec and 8 sec). Error bars indicate the within-subject error across conditions. There was a main effect of condition and duration on confidence rating, but not on meta-cognitive ability. On the right, the BF_10_ from the post-hoc tests on condition are shown. Again, there is a clear oblique effect, with confidence in the oblique condition being worse than in the horizontal and vertical condition. B. The BF_10_ from the post-hoc comparisons of duration on confidence ratings. Dark blue colours indicate more evidence for the null-hypothesis (i.e., no difference in confidence rating between these two durations), while lighter blue to red colours indicate gradual higher evidence for the alternative hypothesis (i.e., evidence for a statistical difference in confidence ratings between the two durations). For interpretation purposes, the colours in the heatmap are log-scaled. The cut-offs for indeterminate evidence ($\frac{1}{3}$ and 3) are shown on the legend by respectively the lower and upper arrow. Overall, confidence is lowest in the shortest and in the longest durations.

Mean confidence over participants and conditions on a 5-point scale was 3.1 (SD = .14), indicating that on average participants felt neutral about the accuracy of their responses: neither very confident nor very unconfident. Mean AROC was .57 (SD = .10), with Bayesian one-sided one sample *t*-tests showing the distributions were higher than .5 (BF_10_ = 929, 161, 6674, and 52 for combined, vertical, horizontal, and oblique motion conditions respectively). Fig. 4A shows the break-down of confidence and AROC over the three conditions and ten durations.

#### 3.2.1 Stimulus duration and motion orientation affect confidence in tactile perception

There was extreme evidence for an effect of condition and of duration on participant confidence, but extreme evidence against an interaction-effect. Similarly to performance, post-hoc tests for condition (Fig. 4A) showed extreme evidence that participants were less confident in the oblique compared to the horizontal and vertical condition, with moderate evidence against a difference between horizontal and vertical. Again, these data suggest that participants were significantly less confident in their perceptual judgements on the oblique axis.

Posthoc tests for the main effect of duration on confidence ratings were also conducted. Due to the large number (35) of tests, the logged BF_10_ are presented as a heatmap in Fig. 4B, with each duration (x-axis) being compared to the other durations (y-axis). For example, the top-left value reflects that there is high evidence that confidence ratings to stimuli lasting 200 msec are statistically lower compared to stimuli lasting 300 msec, as indicated by the high Bayes Factor (BF_10_ > 10,000). Overall, the patterns show confidence was lowest in the very short and the very long tactile stimulus durations - with the highest confidence ratings reported for durations between 680 and 2430 msec.

In contrast, there was extreme evidence against effects of condition, duration, and their interaction on the measure of meta-cognitive ability (AROC). This suggests that participants’ reduced confidence ratings in the oblique condition do not reflect a decline in their sensitivity, but rather match their actual lower performance.

### 3.3 Examining inter-individual differences and task-effects on tactile perception

**Fig. 5:**
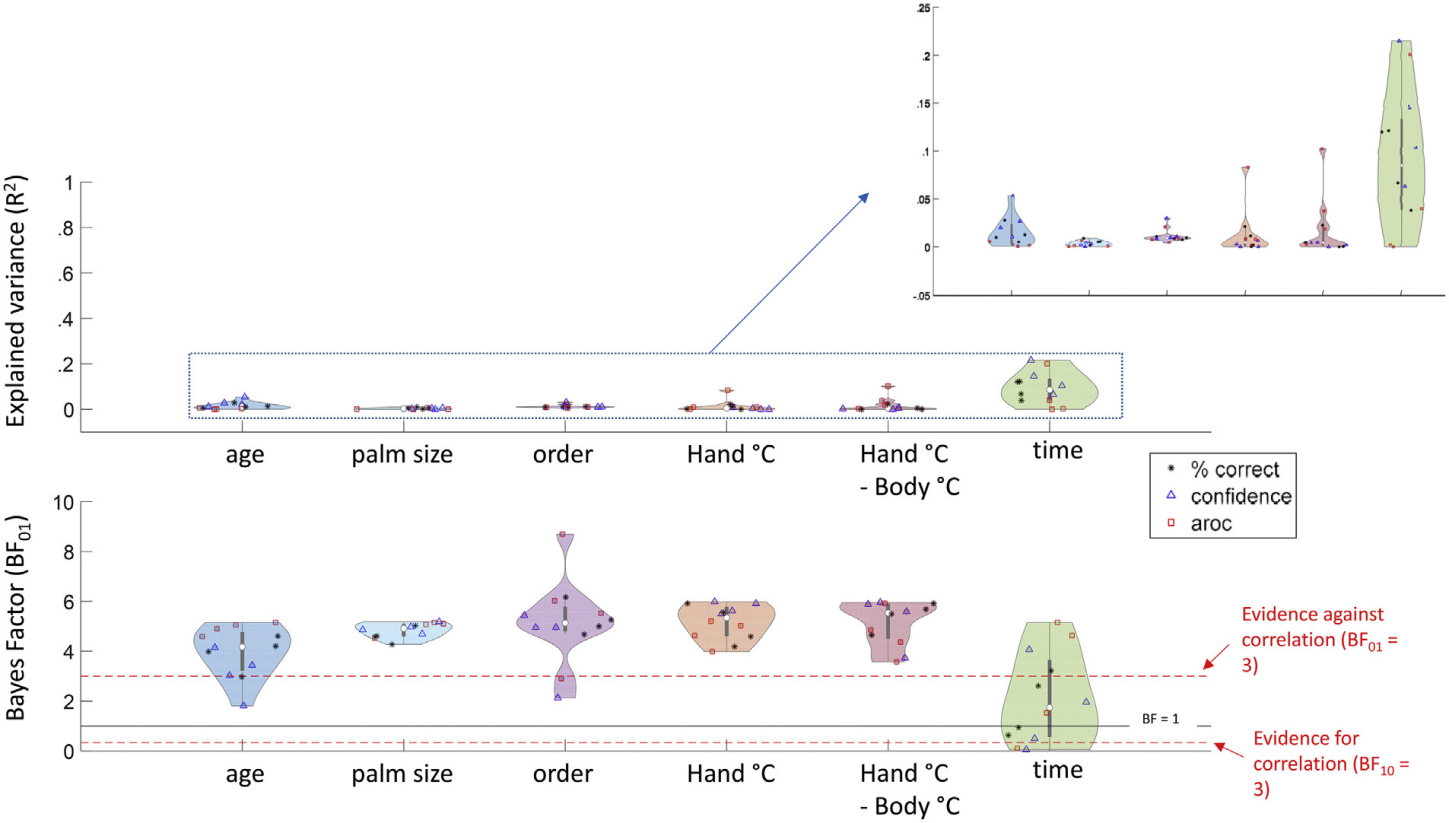
Distributions of the explained variance (R^2^; top panel) and accompanying Bayes Factors (bottom panel) of the correlation analyses and ANOVA of individual differences and task effects. Analyses were conducted for each condition separately as well as combined, with task performance (star), confidence ratings (triangle), and AROC (square) - resulting in 12 R^2^-values in each violin. Analyses are separated between those conducted on the between-subject (left) and on the within-subject level (right). In the bottom panel, values above the upper red line indicate more evidence for the null-hypothesis (BF_01_ > 3). Values between the two red lines are typically interpreted as indeterminate, while evidence below the red line (BF_01_ <$\frac{1}{3}$) indicate evidence for the presence of a correlation/effect. Overall, we find evidence against systematic individual differences and task effects for age, palm size, order, and hand temperature. The evidence for effects of time remain largely indeterminate.

To systematically assess the large number of between- and within-subject, the BF_01_ for each analysis is plotted in violin plots (Fig. 5 - bottom panel). Each within- and between-subject variable is plotted in a separate violin, with each violin showing the 3 measures (accuracy, confidence, and AROC; reflected respectively by an asterisk, a triangle, and a square) × 4 ‘conditions’ (horizontal, vertical, oblique, and combined). Distributions shifted above the top red line show evidence against correlations (or against an effect, for ‘order’). The accompanying explained variance (R^2^) is shown in the top panel. Note that for the within-subject analyses, R^2^ reflects the median of the group distribution. For interpretation purposes, we plotted the symbols on a full range (0–100% explained variance) - reflecting that for all variables except time, effect sizes were negligible across all measures/symbols. For visibility purposes, we also included a ‘zoomed in’ version that shows the measures across a smaller range (but note that small differences between measures may be amplified on such an axis).

Neither performance, confidence, or AROC correlated with the between-subject factors (age and palm size). Likewise, none of the outcome measures were affected by condition order. Within-subject fluctuations in performance, confidence, or AROC were not caused by fluctuations in hand temperature - neither ‘raw’ or normalised by body temperature. Explained variance for all these six variables centered around 0%.

BF_01_ for within-subject correlations between the outcome measures and time were largely indeterminate. Some of the confidence ratings were positively correlated with time - indicating that participants felt more confident in performance as they got more experience. This was, however, not mirrored in their objective performance.

## 4 Discussion

Here we investigated the ability of people to perceive complex whole hand tactile motion stimuli generated using cutting-edge mid-air ultrasound technology. On a fundamental level, we found that participants could discriminate direction above chance level across all motion axes under study, despite no physical contact between the hand and the stimulator. Furthermore, we report evidence of a clear anisotropy in the perception of tactile motion across the palmar surface of the hand. Specifically, performance was poorest for motion discrimination in the oblique axis compared to the horizontal and vertical axes (Fig. 2). The observed pattern was mirrored in measures of subjective confidence: people felt least certain in their motion discrimination judgements when the stimuli were moving in the oblique axis. This finding extends the classic ‘Oblique effect’ (Appelle, 1972) reported in visual motion into the tactile system. By translating the classic studies of motion dot kinetogram from visual to tactile domain, we have provided further clear evidence of commonalities in perceptual biases that transcend sensory modality.

Our results raise new questions regarding the perception of complex tactile percepts that can be projected onto the palm. From a mechanistic perspective: what are the underlying shared processes in the brain that confer common biases in motion perception across differing sensory modalities? From an applications perspective, how can the perceptual predilections of the human brain be used to design effective feedback for touch-free HMIs using mid-air stimulation?

### 4.1 Anisotropy in tactile perception

Anisotropy in tactile perception of orientation has been reported widely on the fingertips. There is with some disagreement regarding the specific axes in which acuity for orientation is highest on the fingertips. Some studies have reported enhanced perception of static tactile grating stimuli when they are oriented in the proximal-distal axis, parallel to the papillary ridges (Essock et al., 1992; Schneider et al., 1986; Vega-Bermudez & Johnson, 2004; Wheat & Goodwin, 2000), however others have reported enhancement in the medial-lateral axis in addition (Lechelt, 1988, 1992), without enhancement in the vertical axis (Bensmaia et al., 2008), or even isotropic perception across all orientations (Craig, 1999). Few studies have considered tactile motion anisotropy. In a study of fingertip motion using a braille pin mounted on a trackpad, evidence for superior perceptual abilities was again reported only in the vertical axis of the fingertip (Keyson & Houtsma, 1995). Reports of direction-dependent perceptual acuity of tactile orientation and motion stimuli have commonly attributed these to the orientation of the skin at the fingertip and differential sensitivity around the tip of the nail.

Here we report an oblique effect on an entirely different scale, with stimuli that extend across the palm of the hand. Using contact free ultrasound methods, we were able to deliver stimuli closely analogous to the random dot kinetograms common in the visual literature. We observe clear evidence of relatively enhanced motion perception in the vertical and horizontal directions aligned with the proximal-distal and medial-lateral axes of the hand. The observation of an oblique effect on this scale is striking, and shows clear distinctions from the more mixed evidence reported by experiments delivering fine-grain stimuli over limited spatial areas at the fingertip. The palm has a much lower receptor density and lower tactile acuity than the fingertips (Johansson & Vallbo, 1979; Mancini et al., 2014), and the cortical representation is correspondingly much smaller (Mountcastle, 2005).

A recent study investigated size perception across the hand, including stimuli along the same three axes as were applied here (Fiori & Longo, 2018). The orientational biases in size perception appeared quite distinct from the evidence of an oblique effect presented herein. While we observed evidence of enhanced motion perception in both the vertical and horizontal axes, this work showed that judgements of size were most accurate when stimuli were presented in the horizontal (medial-lateral) axis and least accurate for stimuli presented in the vertical axis. The authors related this pattern of results to a perceptual stretch model, wherein perceived distance varies sinusoidally as a function of stimulus orientation. Increasing stretch increases the magnitude of the sinusoid, magnifying perceptual biases of size in stimuli shifted away from the horizontal axis. A meta-analysis of similar size perception studies on the palm concluded that an anisotropy exists, such that distances in this axis are perceived as around 10% larger than those in the vertical axis (Longo, 2020). Here we extend on this very recent work to demonstrate that the glabrous skin of the palm, which was previously not thought to show anisotropies in tactile size perception (Longo & Haggard, 2011), also shows a clear pattern of perceptual anisotropy in motion perception.

### 4.2 Neural mechanisms of tactile motion perception

The oblique effect generally appears to be driven by both lower-level *Class-1* and higher level *Class-2* mechanisms in the brain. In the visual domain, *Class-1* mechanisms involve the presence of fewer neurons tuned to oblique orientations in primary visual cortex (V1) compared to those responsive to vertical and horizontal orientations (Essock, 1980; Li et al., 2003), while *Class-2* mechanisms involve higher-level processing, such as memory and learning effects (Essock, 1980).

It is not possible to dissociate *Class-1* and *Class-2* mechanisms of tactile perception from the present design. However, the origins of directional biases in the tactile representations can perhaps be linked to long-term patterns of sensory inputs to the system. Recent work used arrays of up to 30 miniature acceleratometers to measure the patterns of cutaneous vibrations that pass through human hands during single finger, multi-finger, and grasping motions (Shao et al., 2016). This data revealed clear evidence of gradients of vibrational intensity induced sequentially by each movement, which show broad alignment with the cardinal vertical and horizontal axes of the hands. Given the frequency with which we use such movements to interact with the world around us, it seems conceivable that the combination of the anatomy of hand movement, combined with the experience of stereotyped vibrational inputs, shapes the neural tactile representations around the cardinal axes.

The known neural mechanisms of tactile motion perception and its commonalities with primate visual motion processing have been well outlined in two recent reviews (Pack & Bensmaia, 2015; Pei & Bensmaia, 2014). Evidence from primate electrophysiology suggests that Brodmann Area 1 (BA1) in the postcentral gyrus integrates amplitude, direction, and speed information from primary cortical neurons to yield tuning to specific motion directions in relatively larger receptive fields than those observed in other regions of primary somatosensory cortex (S1) (Gardner, 1988; Pei et al., 2010, 2011). In this sense, BA1 seems to subserve a similar function to the middle temporal (MT)/V5 complex in visual motion processing (Pack & Bensmaia, 2015). Evidence from human studies suggests that tactile motion also elicits more widespread activity in anterior intraparietal and inferior parietal areas (Kitada et al., 2003), as well as activation of an area of MT distinct from that implicated in visual motion (Amemiya et al., 2017; Summers et al., 2009; Wacker et al., 2011); however the latter may be an epiphenomenon of visual imagery (Lacey & Sathian, 2011). Directional biases in tactile motion perception appear to be independent of visual input, as tactile perceptual anisotropy has been observed previously in blind individuals (Lechelt, 1988).

### 4.3 Applications of tactile stimuli in touch-free human–machine interfaces

The design of tactile stimuli is still in its infancy compared to other areas of HMI. As tactile technologies advance, so too do the complexity and sophistication of the stimulation methods possible (Schneider et al., 2017). The risk of such rapid advances is that they outpace our understanding of human perception and develop based on the notion that features robustly perceived in one sense can also be perceived in another.

The tactile stimuli under study in this experiment were purposely designed to uncover relative differences across the three motion axes purely in the context of motion, and to avoid a ceiling effect in any one condition, hence their relatively small size (8.5 mm diameter). When comparing receptor densities across the palm and retina, it is unsurprising that overall performance in the perception of these tactile motion ability remained relatively low. Making calculations based on reference densities of rapidly adapting receptor in the palm (.92 receptors/cm^2^) (Johansson & Vallbo, 1979), our .85 cm diameter tactile dots would exautocite fewer than one receptor per frame of movement. In contrast, using reference angular cone density data from the retina, an equivalent visual dot viewed at an equivalent distance (38 cm) would exautocite around 180 cones in the fovea (assuming angular cone density of 180, 000/degree^2^) (Wang et al., 2019) or on average across the entire retinal surface, around 4–5 cones per frame of movement (assuming average angular cone density of 350/degree^2^) (Curcio et al., 1990). On the basis of these figures, it is unsurprising that the stimuli were challenging to perceive, with an overall discrimination performance level of 61% on the group level, while an equivalent visual task would prove simple. Clearly larger non-overlapping dots would activate a large number of peripheral receptors and potentially enhance accuracy using a stimulus that remains purely motion-based.

By studying these challenging stimuli in isolation of complementary features such as orientation or shape, which might implicitly aid motion discrimination judgments, we are able to isolate evidence of the oblique effect. The understanding of this motion detection bias can be applied to more complex composite tactile stimuli used in real world environments, such as HMIs, to enhance user accuracy. The visual oblique effect, characterised similarly in isolated psychophysical experiments, has been reported in a variety of real-world contexts including product design and perception of fine art (Latto et al., 2000; Lidwell et al., 2010).

### 4.4 Time-confidence trade off in complex tactile percepts

Remarkably, we found that accuracy was not affected by stimulus duration, despite the large range of durations used (Table S1). In contrast, raw confidence ratings (but not AROC) were affected, which is a crucial additional consideration in the design of HMIs. Indeed, when thinking about an end user, it is not just important to know if they can a device accurately, but also whether they feel comfortable with it. If a user feels that they are performing poorly and think that perhaps they are unable to understand how the device works, this can lead to discomfort and unwillingness to use said device, even if their objective performance is fine. A device that is suited for its end user should ideally elicit high performance as well as high subjective confidence (i.e., the user feeling confident in their performance) and high metacognitive ability (i.e., the user understanding when they are and are not performing well).

Unexpectedly, the relationship between stimulus duration and confidence assumed a clear non-linear trend (Fig. 4): participants were least confident about the shortest and the longest durations. One explanation may be that longer exposure to the tactile percepts cause desensitisation, leading the perception to become less certain over time. This would decrease confidence, but not necessarily accuracy, if participants stick to their first choice. Our findings are somewhat unexpected, given that previous pilot studies using ultrasound stimuli have employed very long stimulus durations (Rutten et al., 2019). Overall, our results suggest that long exposure to the tactile percepts is at the very minimum unnecessary, but also potentially detrimental to user experience. If this is an issue of desensitisation, in order to apply such ultrasound techniques to HMIs, there would be a clear advantage to selecting stimulus features that take advantage of perceptual biases (e.g., brief vertical motion) to enhance the accuracy of perceived feedback.

### 4.5 Applications involving shapes

To our knowledge, this study is the first to rigorously test for the feasibility of whole hand tactile perception using ultrasound stimuli. To date, the majority of studies applying this technology have been usability pilots, which have focused on the parallels between ultrasound stimuli and a visual screen or display. As a result, most of this work has focused on shapes: a visual feature that appears intuitive to translate into the tactile domain. These pilot studies were proof-of-concept, and therefore employed limited sample sizes and/or small trials numbers, which preclude the use inferential statistics, limiting interpretability. However, this literature provides a foundation for the present study, and our desire to focus on motion rather than shape as a tactile feature for whole hand perception.

A recent example was a study testing fifteen participants in a shape-discrimination task (Korres and Eid, 2016). On each trial, one of the four possible stimuli (line, circle, triangle, plus-sign) was presented for maximum 30 sec, with the task consisting of 24 trials in total. Accuracy on the group level was highly variable across shapes (44–76%) - though these are difficult to interpret because each trial featured all stimuli as options. For example, the line stimulus was recognised correctly in 44% of trials, which is clearly above chance level, but it was misidentified as a circle in 51% of trials - which is concerning given the obvious spatial differences between lines and circles. Furthermore, reaction times (RT) were very slow ($\bar{RT}$ = 13.9 sec over trials and participants). Before the experimental trials there was an unlimited period of training (times not reported), clouding interpretation of the results. Another pilot study (Rutten et al., 2019) did not include any training, aiming to measure baseline performance. They tested a similar discrimination-task on 50 participants, with eight different stimuli (four static, four moving) that were presented for 9 sec maximum per trial. The experiment consisted of 40 trials (5 blocks, each consisting of one trial for each stimulus). Accuracy was low to moderate (26–60% on group level across stimuli). It should be noted that their random-without-replacement design may produce progressive determination effects, meaning participants explicitly take their choice on trial *n-1* into account for their choice of trial *n*, making it difficult to determine chance level (Blais, 2008).

Other work has considered the application of virtual 3-D shapes using ultrasound (Long et al., 2014), asking participants to discriminate between five shapes (sphere, pyramid, horizontal prism, vertical prism, and cube), and found mean accuracy scores between 66.7% and 94.4% across shapes. Indeed, the exploring of edges seems more in line with the way we use our hands in daily life. Again however, power was low (6 participants with 15 trials each), and participants had an unlimited training period, necessitating further testing to definitively compare the perception of 2D versus 3D tactile shapes generated with ultrasound.

Although shape discrimination using active touch draws intuitive parallels between the tactile and visual system, this specific sensory feature may not be best suited for rapidly conveying sensory information via the hand. Shape discrimination relies on haptic exploration of a virtual object meaning motor behaviour accounts for participant variance. In contrast, motion stimuli targeted to the hand using infra-red tracking provide a greater degree of control over delivery, rendering them a more appealing mechanism for the rapid feedback required in touch-free interactions with HMIs (Breitschaft et al., 2019). Importantly, tactile motion stimuli could also be integrated into touch interfaces that are not touch-free, for example, via actuators embedded in car steering wheels or clothing.

### 4.6 Limitations and future directions

While the question of feasibility and accuracy at the group level is important, the performance of *individual* participants in perceiving complex tactile percepts is relevant both from a mechanistic perspective (uncovering neurobiological processes) and from an applied perspective (testing feasibility for specific user-groups). We found large individual differences in performance that were not explained by our candidate variables. The lack of an observed relationship between performance and age likely resulted from a relatively young participant group. Tactile sensitivity in the fingertips is known to decrease with age and to be affected by gender, necessitating further evaluation of the accessibility of HMIs that result on ultrasound feedback (Goldreich & Kanics, 2003; Stevens et al., 1996; Thornbury & Mistretta, 1981).

In our current design, each trial consisted of two stimuli, one for each direction along the conditional axis. It therefore remains unknown whether differences in performance can be found in motion discrimination along the same axis (e.g., whether one is better at distinguishing up motions compared to down motions). Future work may present trials with only a single stimulus per trial, which would make such differentiation possible. Such a design would also allow for the proper measurement of reaction times. More pressingly though, because conditions were presented in a blocked order, participants had clear expectations on the directions the two stimuli would move to. While these expectations are unlikely to affect the presence of the found oblique effect (as this is true for all conditions), it is very likely that group distributions of discrimination performance would be lower if participants do not know beforehand along which axis they will have to discriminate. Indeed, it is known that expectations can increase (tactile) perceptual performance (van Ede et al., 2010, 2011), potentially by modulating oscillatory activity in S1. Future experiments with single-stimulus trials may include spatial-attentional cues to investigate these effects further.

From an applied perspective, one open question relates to the immobilisation of participants’ movement and immobilisation. In the current set-up, arms were immobilised with an arm rest and velcro straps. As the support only went up until the wrist, participants had to put continuous motor effort into keeping their hands parallel to the device (though not as much as without the support). Previous research has found that tactile detection performance at a body part decreases when the stimulus is presented just before or during a movements of said body part compared to rest (Chapman & Beauchamp, 2006; Voss et al., 2008; Voudouris & Fiehler, 2017; Williams & Chapman, 2002). This decrease is not only found with active movement, but also when the body part is passively moved (Chapman & Beauchamp, 2006), and is known to relate specifically to movement planning rather than to the movement itself (Voss et al., 2008). Importantly though, tactile suppression effect is context-dependent: tactile sensitivity may instead heighten when this is advantageous for the task (e.g., Voudouris & Fiehler (2017)). It remains speculative how these suppression and enhancement effects caused by planning of discrete movements relate to a continuous motor plan to keep the hand as still as possible. Future studies may take quantitative measures of participant movement to relate these to performance and meta-cognition at a within-subject level.

As a positive consequence of the current immobilisation, participants’ hands and arms were kept in a straight line at all times, which means that the stimuli moved in the directions they were supposed to move along (e.g., in the vertical conditions, the stimuli would indeed move from the bottom to the top of the palm and vice versa). As such, we can rule out any potential within-subject effects of hand posture on the current results. This is certainly not trivial, as research on tactile *localisation* has consistently shown that body posture impacts tactile perception. To localise a tactile stimulus, one must code where the stimulus is on the body (somatotopic reference frame), but also code information about the position of that body part in external space (body-centered or external reference frame). The relevance of this becomes clear when the two reference frames carry contradictory information - for example, when one crosses their arms and their right hand is located on the left side of space - leading to decreased localisation performance (Azañón et al., 2015; Heed & Azañón, 2014; Overvliet et al., 2011; Yamamoto & Kitazawa, 2001). It is not unlikely that similar conflicts and subsequent decreased performance may arise in motion discrimination when body posture is altered such that motion along the axis of the hand is no longer congruent with motion compared to the axis of the rest of the body - though this remains an empirical question. Whether alterations in body posture would also affect conditional differences is a more speculative question, and may partially relate to the potential *Class-1* and *Class-2* mechanisms of the tactile oblique effect (e.g., very low-level skin-receptor features, which would remain stable regardless of hand orientation, versus higher abstract reference frames, which may be more affected by posture).

Another question of interest is to what extent whole hand tactile perception can improve with training. We found evidence against improvement in performance over time. However, aside from a few training trials, participants did not receive any feedback on their answers throughout. This could explain why some of our participants scored below chance level: they may have felt a difference between the two stimuli, but mislearned the association between stimulus and direction. Previous work on visual motion perception has found that training effects are usually limited. For example, training visual motion discrimination along a particular axis can improve performance, but this improvement does not carry over to performance along new axes (Ball & Sekuler, 1987). This means that even if participants can learn whole hand motion discrimination with feedback, it is doubtful that this will show transfer effects to other tasks or even motion directions. The potential lack of a transfer effect will depend heavily on the end user. For example, in the context of users with sensory impairment, the prospect of prolonged training to learn individual stimulus types might be acceptable. In contrast, in the context of commercial HMIs in cars and clinical settings, such a learning curve would be less realistic. A more fruitful future approach may be to examine cross- rather than intra-modality training effects - training on visual and testing on tactile, or vice versa, to tap into the multisensory nature of perception.

## 5 Conclusion

The current study is the first to investigate the perception of the whole hand complex tactile stimuli that have been made feasible with ultrasound techniques. In spite of the relatively sparse innervation of the palm compared with the fingertips, we found participants were able to perceive subtle moving dot stimuli above chance level. Using these stimuli were found clear evidence of an oblique effect in the perception of tactile motion across the hand. Motion aligned with the cardinal horizontal and vertical axes of the hand was perceived significantly more easily and confidently than that aligned with an oblique axis. In addition, participants felt most confident in the perception of stimuli around 500–2500 msec in duration.

A robust understanding for the perceptual biases in these complex tactile percepts will advance the implementation of touch-free tactile interfaces in practical contexts such as accessibility (e.g., haptic aids for visually impaired patients) and safety critical user interfaces (e.g., reducing visual overload in cars). The potential for mid-air tactile feedback to improve the accuracy of touch-free HMIs in clinical settings and busy public environments is also an attractive future application in the context of reducing the transmission of communicable diseases (Otter & French, 2009; Rossol et al., 2014). However, such uses should avoid the temptation to directly translate stimuli, such as shape, from the visual domain into the tactile, albeit technically feasible. While we demonstrate that biases such as the oblique effect exist across sensory boundaries, vision and touch have unique predilections and acuities that, once identified, can be leveraged for practical purposes in HMIs.

## Author contributions

**Marlou Nadine Perquin**: Conceptualisation, Methodology, Software, Formal Analysis, Investigation, Writing, Data Curation, Visualisation. **Mason Taylor**: Formal Analysis, Investigation, Data Curation, Writing - Review & Editing. **Jarred Lorusso**: Conceptualisation, Methodology, Software, Formal Analysis, Investigation, Data Curation, Writing - Review & Editing. **James Kolasinski**: Conceptualisation, Methodology, Software, Writing, Visualisation, Supervision, Funding Acquisition.

## Open practices

The study in this article earned an Open Data badge for transparent practices. Data for this study can be found at https://osf.io/v89tw/?view_only=0a820f68050c44999200a2461b79b636.

The raw data and analyses have been made publicly available on https://osf.io/v89tw/. The study materials will be added to this OSF folder as well when our labs are accessible again, after the covid-related lockdown is over.

## Figures and Tables

**Table S1:** Overview of the mean (SD) of % correct over the different durations, for each of the three conditions and for all conditions combined.

Duration	Combined	Vertical	Horizontal	Oblique
D1	.60 (.11)	.62 (.14)	.60 (.14)	.57 (.13)
D2	.61 (.13)	.61 (.17)	.61 (.15)	.60 (.16)
D3	.62 (.13)	.62 (.19)	.62 (.15)	.61 (.16)
D4	.60 (.16)	.60 (.19)	.61 (.18)	.60 (.16)
D5	.61 (.13)	.64 (.15)	.61 (.16)	.58 (.17)
D6	.60 (.15)	.61 (.18)	.60 (.18)	.59 (.16)
D7	.61 (.18)	.63 (.20)	.64 (.21)	.58 (.19)
D8	.60 (.16)	.62 (.21)	.61 (.18)	.56 (.18)
D9	.61 (.17)	.62 (.20)	.63 (.20)	.58 (.20)
D10	.61 (.17)	.65 (.19)	.62 (.21)	.57 (.21)

## Supplementary results

The Bayesian RM ANOVA on % correct with condition and duration as independent factors showed extremely strong evidence that participants’ accuracy was not dependent on the duration of the stimulus (BF_01_ = 14,184) = see Supplementary Table S1 for all the means and standard deviations of accuracy over the different conditions.

## References

[bib22] Abdouni A., Clark R., Georgiou O. (2019). 2019 International Conference on Multimodal Interaction. ICMI ’19.

[bib56] Amemiya T., Beck B., Walsh V., Gomi H., Haggard P. (2017). Visual area V5/hMT+ contributes to perception of tactile motion direction: A TMS study. Scientific Reports.

[bib13] Appelle S. (1972). Perception and discrimination as a function of stimulus orientation: The “oblique effect” in man and animals. Psychological Bulletin.

[bib75] Azañón E., Stenner M.-P., Cardini F., Haggard P. (2015). Dynamic tuning of tactile localization to body posture. Current Biology.

[bib14] Ball K., Sekuler R. (1980). Models of stimulus uncertainty in motion perception. In: Psychological Review.

[bib15] Ball K., Sekuler R. (1987). Direction-specific improvement in motion discrimination. Vision Research.

[bib42] Bensmaia S.J., Hsiao S.S., Denchev P.V., Killebrew J.H., Craig J.C. (2008). The tactile perception of stimulus orientation. Somatosensory & Motor Research.

[bib63] Blais C. (2008). Random without replacement is not random: Caveat emptor. Behavior Research Methods.

[bib65] Breitschaft S.J., Clarke S., Carbon C.-C. (2019). A theoretical framework of haptic processing in automotive user interfaces and its implications on design and engineering. Frontiers in Psychology.

[bib1] Carter T., Seah S.A., Long B., Drinkwater B., Subramanian S. (2013). Proceedings of the 26th annual ACM symposium on user interface software and technology. UIST ’13.

[bib71] Chapman C.E., Beauchamp E. (2006). Differential controls over tactile detection in humans by motor commands and peripheral reafference. Journal of Neurophysiology.

[bib7] Cody F.W.J., Garside R.A.D., Lloyd D., Poliakoff E. (2008). Tactile spatial acuity varies with site and axis in the human upper limb. Neuroscience Letters.

[bib43] Craig J.C. (1999). Grating orientation as a measure of tactile spatial acuity. Somatosensory & Motor Research.

[bib60] Curcio C.A., Sloan K.R., Kalina R.E., Hendrickson A.E. (1990). Human photoreceptor topography. The Journal of Comparative Neurology.

[bib48] Essock E.A. (1980). The oblique effect of stimulus identification considered with respect to two classes of oblique effects. Perception.

[bib37] Essock E.A., Krebs W.K., Prather J.R. (1992). An anisotropy of human tactile sensitivity and its relation to the visual oblique effect. Experimental Brain Research.

[bib11] Fiori F., Longo M.R. (2018). Tactile distance illusions reflect a coherent stretch of tactile space. Proceedings of the National Academy of Sciences.

[bib32] Fleming S.M., Lau H.C. (2014). How to measure metacognition. Frontiers in Human Neuroscience.

[bib31] Fleming S.M., Weil R.S., Nagy Z., Dolan R.J., Rees G. (2010). Relating introspective accuracy to individual differences in brain structure. Science.

[bib2] Gardner E.P. (1988). Somatosensory cortical mechanisms of feature detection in tactile and kinesthetic discrimination. Canadian Journal of Physiology and Pharmacology.

[bib66] Goldreich D., Kanics I.M. (2003). Tactile acuity is enhanced in blindness. The Journal of Neuroscience.

[bib76] Heed T., Azañón E. (2014). Using time to investigate space: A review of tactile temporal order judgments as a window onto spatial processing in touch. Frontiers in Psychology.

[bib10] Hidaka S., Tamè L., Zafarana A., Longo M.R. (2020). Anisotropy in tactile time perception. Cortex.

[bib30] JASP Team (2019).

[bib45] Johansson R.S., Vallbo A.B. (1979). Tactile sensibility in the human hand: Relative and absolute densities of four types of mechanoreceptive units in glabrous skin. In: The Journal of Physiology.

[bib27] Kennet S., Taylor-Clarke M., Haggard P. (2001). Noninformative vision improves the spatial resolution of touch in humans. Current Biology.

[bib44] Keyson D.V., Houtsma A.J. (1995). Directional sensitivity to a tactile point stimulus moving across the fingerpad. Perception & Psychophysics.

[bib53] Kitada R., Kochiyama T., Hashimoto T., Naito E., Matsumura M. (2003). Moving tactile stimuli of fingers are integrated in the intraparietal and inferior parietal cortices. Neuroreport.

[bib9] Knight F.L.C., Longo M.R., Bremner A.J. (2014). Categorical perception of tactile distance. Cognition.

[bib24] Korres G., Eid M. (2016). Ultrasonic point-cloud tactile stimulation. IEEE Access.

[bib57] Lacey S., Sathian K. (2011). Multisensory object representation: Insights from studies of vision and touch. Progress in Brain Research.

[bib61] Latto R., Brain D., Kelly B. (2000). An oblique effect in aesthetics: Homage to Mondrian (1872-1944). Perception.

[bib40] Lechelt E.C. (1988). Spatial asymmetries in tactile discrimination of line orientation: A comparison of the sighted, visually impaired, and blind. Perception.

[bib41] Lechelt E.C. (1992). Tactile spatial anisotropy with static stimulation. Bulletin of the Psychonomic Society.

[bib34] Lee M.D., Wagenmakers E.-J. (2013).

[bib62] Lidwell W., Holden K., Butler J. (2010).

[bib49] Li B., Peterson M.R., Freeman R.D. (2003). Oblique effect: A neural basis in the visual cortex. Journal of Neurophysiology.

[bib47] Longo Matthew R. (2020). Tactile distance anisotropy on the palm: A meta-analysis. Attention, Perception & Psychophysics.

[bib8] Longo M.R., Haggard P. (2011). Weber's illusion and body shape: Anisotropy of tactile size perception on the hand. Journal of Experimental Psychology. Human Perception and Performance.

[bib64] Long B., Seah S.A., Carter T., Subramanian S. (2014). Rendering volumetric haptic shapes in mid-air using ultrasound. ACM Transactions on Graphics.

[bib46] Mancini F., Bauleo A., Cole J., Lui F., Porro C.A., Haggard P., Iannetti G.D. (2014). Whole-body mapping of spatial acuity for pain and touch. Annals of Neurology.

[bib29] MATLAB (2019).

[bib23] Mizobuchi K., Kuwabara S., Toma S., Nakajima Y., Ogawara K., Hattori T. (2000). Single unit responses of human cutaneous mechanoreceptors to air-puff stimulation. Clinical Neurophysiology.

[bib6] Mountcastle V.B. (2005).

[bib12] Murray M.M., Lewkowicz D.J., Amedi A., Wallace M.T. (2016). Multisensory processes: A balancing act across the lifespan. Trends in Neurosciences.

[bib26] Oldfield R.C. (1971). The assessment and analysis of handedness: The Edinburgh Inventory. Neuropsychologia.

[bib79] Otter J.A., French G.L. (2009). Bacterial contamination on touch surfaces in the public transport system and in public areas of a hospital in London. Letters in Applied Microbiology.

[bib77] Overvliet K.E., Azañón E., Soto-Faraco S. (2011). Somatosensory saccades reveal the timing of tactile spatial remapping. Neuropsychologica.

[bib52] Pack C.C., Bensmaia S.J. (2015). Seeing and feeling motion: Canonical computations in vision and touch. PLoS Biology.

[bib51] Pei Y.-C., Bensmaia S.J. (2014). The neural basis of tactile motion perception. Journal of Neurophysiology.

[bib3] Pei Y.-C., Hsiao S.S., Craig J.C., Bensmaia S.J. (2010). Shape invariant coding of motion direction in somatosensory cortex. PLoS Biology.

[bib4] Pei Y.-C., Hsiao S.S., Craig J.C., Bensmaia S.J. (2011). Neural mechanisms of tactile motion integration in somatosensory cortex. Neuron.

[bib18] Peirce J.W. (2007). PsychoPy—psychophysics software in python. Journal of Neuroscience Methods.

[bib19] Peirce J.W. (2009). Generating stimuli for neuroscience using PsychoPy. Front. Neuroinformat..

[bib20] Peirce J.W., Gray J.R., Simpson S., MacAskill M., Höchenberger R., Sogo H., Kastman E., Lindeløv J.K., PsychoPy2 “ (2019). Experiments in behavior made easy. Behavior Research Methods.

[bib80] Rossol N., Cheng I., Shen R., Basu A. (2014). Conference proceedings: ... Annual International Conference of the IEEE Engineering in Medicine and Biology Society. IEEE Engineering in Medicine and Biology Society. Annual Conference 2014.

[bib16] Rouder J.N. (2014). Optional stopping: No problem for Bayesians. Psychonomic Bulletin & Review.

[bib25] Rutten I., Frier W., van der Bogaert L., Geerts D. (2019). Extended Abstracts of the 2019 CHI Conference on Human Factors in Computing Systems LBW0283.

[bib36] Schneider, Hughes B., Epstein W., Bach-y-Rita P. (1986). The detection of length and orientation changes in dynamic vibrotactile patterns. Perception & Psychophysics.

[bib58] Schneider O., MacLean K., Swindells C., Booth K. (2017). Haptic experience design: What hapticians do and where they need help. International Journal of Human-Computer Studies. Multisensory Human-Computer Interaction.

[bib17] Schönbrodt F.D., Wagenmakers E.-J., Zehetleitner M., Perugini M. (2017). Sequential hypothesis testing with Bayes factors: Efficiently testing mean differences. Psychological methods.

[bib50] Shao Y., Hayward V., Visell Y. (2016). Spatial patterns of cutaneous vibration during whole-hand haptic interactions. Proceedings of the National Academy of Sciences.

[bib67] Stevens J.C., Foulke E., Patterson M.Q. (1996). Tactile acuity, aging, and braille reading in long-term blindness. Journal of Experimental Psychology: Applied.

[bib54] Summers I.R., Francis S.T., Bowtell R.W., McGlone F.P., Clemence M. (2009). A functional-magnetic-resonance-imaging investigation of cortical activation from moving vibrotactile stimuli on the fingertip. The Journal of the Acoustical Society of America.

[bib5] Tamè L., Tucciarelli R., Sadibolova R., Sereno M.I., Longo M.R. (2019). Reconstructing neural representations of tactile space. bioRxiv.

[bib28] Taylor-Clarke M., Kennett S., Haggard P. (2002). Vision modulates somatosensory cortical processing. Current Biology.

[bib68] Thornbury J.M., Mistretta C.M. (1981). Tactile sensitivity as a function of age. Journal of Gerontology.

[bib70] van Ede F., de Lange F., Jensen O., Maris E. (2011). Orienting attention to an upcoming tactile event involves a spatially and temporally specific modulation of sensorimotor alpha- and beta-band oscillations. Journal of Neuroscience.

[bib69] van Ede F., Jensen O., Maris E. (2010). Tactile expectation modulates pre-stimulus beta-band oscillations in human sensorimotor cortex. Neuroimage.

[bib39] Vega-Bermudez F., Johnson K.O. (2004). Fingertip skin conformance accounts, in part, for differences in tactile spatial acuity in young subjects, but not for the decline in spatial acuity with aging. Perception & Psychophysics.

[bib72] Voss M., Ingram J.N., Wolpert D.M., Haggard P. (2008). Mere expectation to move causes attenuation of sensory signals. PLoS ONE.

[bib73] Voudouris D., Fiehler K. (2017). Enhancement and suppression of tactile signals during reaching. Journal of Experimental Psychology: Human Perception and Performance.

[bib55] Wacker E., Spitzer B., Lützkendorf R., Bernarding J., Blankenburg F. (2011). Tactile motion and pattern processing assessed with high- field fMRI. PLoS ONE.

[bib33] Wagenmakers E.-J., Marsman M., Jamil T., Ly A., Verhagen J., Love J., Selker R., Gronau Q.F., Šmíra M., Epskamp S., Matzke S., Rouder J.N., Morey R.D. (2018). Bayesian inference for psychology. Part I: Theoretical advantages and practical ramifications. Psychonomic Bulletin & Review.

[bib35] Wagenmakers E.-J., Love J., Marsman M., Jamil T., Ly A., Verhagen J., Selker R., Gronau Q.F., Dropmann D., Boutin B., Meerhoff F., Knight P., Raj P., van Kesteren E.-J., van Doorn J., Šmíra M., Epskamp S., Etz A., Matzke D., Morey R.D. (2018). Bayesian inference for psychology. Part II: Example applications with JASP. Psychonomic Bulletin & Review.

[bib59] Wang Y., Bensaid N., Tiruveedhula P., Ma J., Ravikumar S., Roorda A. (2019). Human foveal cone photoreceptor topography and its dependence on eye length. eLife.

[bib21] Weichert F., Bachmann D., Rudak B., Fisseler D. (2013). Analysis of the accuracy and robustness of the Leap motion controller. Sensors (Basel, Switzerland).

[bib38] Wheat H.E., Goodwin A.W. (2000). Tactile discrimination of gaps by slowly adapting afferents: Effects of population parameters and anisotropy in the fingerpad. Journal of Neurophysiology.

[bib74] Williams S.R., Chapman C.E. (2002). Time course and magnitude of movement-related gating of tactile detection in humans. III. Effect of motor tasks. Journal of Neurophysiology.

[bib78] Yamamoto S., Kitazawa S. (2001). Reversal of subjective temporal order due to arm crossing. Nature Neuroscience.

